# Rhizospheric Microbiome Profiling of *Capsicum annuum* L. Cultivated in Amended Soils by 16S and Internal Transcribed Spacer 2 rRNA Amplicon Metagenome Sequencing

**DOI:** 10.1128/genomeA.00626-17

**Published:** 2017-07-27

**Authors:** Ali Asaff-Torres, Mariana Armendáriz-Ruiz, Manuel Kirchmayr, Raúl Rodríguez-Heredia, Marcos Orozco, Juan Carlos Mateos-Díaz, Luis Figueroa-Yáñez, Itzamná Baqueiro-Peña, Jorge Verdín

**Affiliations:** a Innovak Global, Chihuahua, Chihuahua, Mexico; b CIATEJ-Centro de Investigación y Asistencia en Tecnología y Diseño del Estado de Jalisco, A.C., Zapopan, Jalisco, Mexico; c CIAD-Centro de Investigación en Alimentación y Desarrollo, A.C., Hermosillo, Sonora, Mexico; d CONACyT, CIAD-Centro de Investigación en Alimentación y Desarrollo, A.C., Hermosillo, Sonora, Mexico

## Abstract

Rhizospheric microbiomes of *Capsicum annuum* L. cultivated either conventionally or amended with a synthetic microbial consortium or a root exudate inductor, were characterized by 16S/internal transcribed spacer 2 (ITS2) rRNA amplicon metagenome sequencing. The most abundant taxa found, although differently represented in each treatment, were *Gammaproteobacteria*, *Alphaproteobacteria*, *Actinobacteria*, and *Bacilli*, as well as Chytridiomycetes and Mortierellomycotina.

## GENOME ANNOUNCEMENT

Rhizospheric microbial communities influence plant development, growth, and health (1–3). Higher crop yields are achievable if beneficial microbiota are used to enrich the plant rhizosphere (2, 4). Amendment of soils with synthetic microbial consortia allows the introduction of specific beneficial microbiota into the root rhizosphere. Alternatively, the application of root exudate inductors aims to stimulate root natural microbiota-recruiting mechanisms (5–7). Here, rhizosphere and rhizoplane microbiomes from *Capsicum annuum* L. cultivated by conventional farming, soil amended with a synthetic microbial consortium (BioFit RTU; Innovak Global, Mexico), and a phenolics-based root exudate inductor (ExuRoot; Innovak Global) were investigated by 16S and internal transcribed spacer 2 (ITS2) rRNA amplicon metagenome analysis.

*C. annuum* L. was cultivated in Chihuahua, Mexico (28°42′10″N, 105°57′42″W), under open-field conditions from May to June 2016. BioFit RTU and ExuRoot amendments were applied five times at days 7, 14, 24, 35, and 46 posttransplantation (4.0 and 3.72 kg/ha/application, respectively). Just after first flowering, 20 plants/replicate, with two replicates from each of three treatments were sampled, in addition to duplicated control samples consisting of a 19-cm column of bulk soil taken 2 cm beneath the surface. Rhizospheres (root tightly bound soil) from each plant/treatment were extracted with phosphate-buffered saline (PBS)-Silwet MAXX (0.02% [vol/vol]) (Arysta Life Science, Mexico) and pooled. Rhizoplanes from each plant/treatment were obtained by sonication (160 W, 30 s for 10 cycles) of roots after rhizosphere extraction and pooled. Rhizosphere and rhizoplane metagenomic DNA was extracted with the PowerSoil DNA isolation kit (Mo Bio, Inc., Carlsbad, CA), and its integrity and concentration were assessed by agarose gel electrophoresis and UV spectroscopy. 16S V3-V4 and ITS2 rRNA genes were amplified with primers 337F/805R (8) and ITS3F/ITS4R (9), respectively.

Amplicon metagenomic libraries were paired-end sequenced on an Illumina MiSeq platform. A total of 10,511,842 raw reads were obtained for the 16S libraries, and 11,667,652 raw reads were obtained for ITS2 ones. Sequencing reads were analyzed and processed with CLC Genomics Workbench 9.0 supplemented with CLC Microbial Genomics Module 1.3 (Qiagen, Denmark). Raw reads were overlapped into single longer reads and fixed-length trimmed; chimeras and reads showing <100 abundance were removed. To identify operational taxonomic units (OTUs), from 84,259 to 141,161 filtered reads from 16S libraries and 829 to 17,235 reads from ITS2 ones were clustered using the reference databases SILVA 16S version 123 (10) and UNITE version 7.1 (11), with 97% identity as the clustering criterion. A total of 9,644 OTU were predicted for 16S libraries, and 43 OTU were predicted for ITS2 libraries.

The most abundant bacterial classes in both bulk soil and rhizosphere/rhizoplane samples from all three farming conditions were *Gammaproteobacteria*, *Alphaproteobacteria*, *Actinobacteria*, *Bacilli*, and *Planctomycetacia*. *Gammaproteobacteria* and *Bacilli* were enriched in rhizosphere and rhizoplane samples under all farming conditions compared to bulk soil ones. Chytridiomycetes, Agaromycetes, and Mortierellomycotina were the most abundant fungal classes found. Compared to bulk soil, Agaromycetes were reduced in the rhizosphere and rhizoplane from conventionally farmed and ExuRoot-amended plants, whereas Mortierellomycotina abundances were reduced only in BioFit-amended plants.

### Accession number(s).

The sequences obtained in this study were made public in the Sequence Read Archive (SRA) via the National Center for Biotechnology Information (NCBI) under the accession number SRP107093.
